# Adolescent fiber intake and mammographic breast density in premenopausal women

**DOI:** 10.1186/s13058-016-0747-8

**Published:** 2016-08-12

**Authors:** Lusine Yaghjyan, Gabriela L. Ghita, Bernard Rosner, Maryam Farvid, Kimberly A. Bertrand, Rulla M. Tamimi

**Affiliations:** 1 Department of Epidemiology, University of Florida, College of Public Health and Health Professions and College of Medicine, 2004 Mowry Rd., Gainesville, 32610 FL USA; 2 Department of Biostatistics, University of Florida, College of Public Health and Health Professions and College of Medicine, Gainesville, FL USA; 3 Channing Division of Network Medicine, Department of Medicine, Brigham and Women’s Hospital and Harvard Medical School, Boston, MA USA; 4 Department of Nutrition, Harvard T.H. Chan School of Public Health, Boston, MA USA; 5 Harvard/Massachusetts General Hospital Center on Genomics, Vulnerable Populations, and Health Disparities, Mongan Institute for Health Policy, Massachusetts General Hospital, Boston, MA USA; 6 Slone Epidemiology Center at Boston University, Boston, MA USA

**Keywords:** Adolescent diet, Breast density, Fiber intake

## Abstract

**Background:**

To date, there is limited and inconsistent epidemiologic evidence for associations of adolescent diet with mammographic breast density, a strong and consistent predictor of breast cancer. We investigated the association of adolescent fiber intake with mammographic density in premenopausal women.

**Methods:**

This study included 743 cancer-free premenopausal women (mean age, 44.9 years) within the Nurses’ Health Study II cohort. Percent breast density, absolute dense and non-dense areas were measured from digitized film mammograms using a computer-assisted thresholding technique. Adolescent and adult diet were assessed with a food frequency questionnaire; energy-adjusted nutrient intakes were estimated for each food item. Information regarding breast cancer risk factors was obtained from baseline or biennial questionnaires closest to the mammogram date. We used generalized linear regression to quantify associations between quartiles of adolescent fiber intake and each of the breast density measures, adjusted for potential confounders. Associations were examined separately for total fiber intake; fiber from fruits, vegetables, legumes, and cereal; and food sources of fiber (fruits, vegetables, and nuts).

**Results:**

In multivariable analyses, total fiber intake during adolescence was not associated with percent breast density (*p* for trend = 0.64), absolute dense area (*p* for trend = 0.80), or non-dense area (*p* for trend = 0.75). Similarly, neither consumption of fiber from fruits, vegetables, legumes, or cereal nor specific sources of fiber intake (fruits, vegetables, or nuts) during adolescence were associated with any of the mammographic density phenotypes.

**Conclusions:**

Our findings do not support the hypothesis that adolescent fiber intake is associated with premenopausal mammographic breast density.

## Background

Mammographic breast density is a well-established and strong predictor of breast cancer risk [1–4]. Appearance of the breast on the mammogram is a reflection of the amount of fat, connective tissue, and epithelial tissue in the breast [3]. Light (non-radiolucent) areas on the mammogram represent the fibrous and glandular tissues (“mammographically dense”), whereas, the dark (radiolucent) areas are primarily fat. Women with breasts of 75 % or greater percent density (proportion of the total breast area that appears dense on the mammogram) are at four- to sixfold greater risk of breast cancer compared to women with more fat tissues in the breasts [3, 5, 6]. Absolute dense area of the breast that represents fibroglandular tissue has been shown to be positively associated with breast cancer risk in both pre- and postmenopausal women [7–13], while non-dense area of the breast (representing adipose tissue) has been inversely associated with breast cancer risk [7, 9, 14, 15].

Some previous studies have suggested that higher fiber intake, particularly during earlier life, may be associated with a reduced risk of breast cancer later in life [16, 17]. To date, there is limited epidemiologic evidence that diet has a strong influence on mammographic density and inconsistency in results for specific dietary factors [18–24]. An earlier study by Brisson et al. demonstrated an inverse association between adult fiber intake and breast density [25], while a more recent study by Vachon et al. did not find statistically significant associations [22]. Adolescence is a time period of rapid growth and development and emerging evidence suggests that exposures during this time period, including nutrition, may be particularly relevant to breast cancer risk [16, 26–30], which could potentially be reflected in the degree of breast density. However, only a few studies investigated the association between adolescent diet and adult breast density [31–35]. Further, none of these studies comprehensively investigated associations of total fiber intake and fiber from specific sources with breast density. We examined associations of adolescent fiber intake with percent breast density, absolute dense and non-dense areas in premenopausal women using prospective data in healthy women from the Nurses’ Health Study II. These associations were examined separately for total fiber intake; fiber from fruits, vegetables, legumes, and cereal; and food sources of fiber (fruits, vegetables, and nuts).

## Methods

### Study population and design

Women included in this study were selected from participants of a breast cancer case-control study nested within the Nurses’ Health Study II (NHS II) cohort. This prospective cohort was established in 1989 and followed 116,430 female registered nurses in the United States who were 25–42 years old (NHS II) at enrollment. After administration of the initial questionnaire, information on breast cancer risk factors (body mass index [BMI], reproductive history, and alcohol use) and any diagnoses of cancer or other diseases was updated through biennial questionnaires [3, 36].

A nested case-control approach was originally used as an efficient design to examine the association between selected biomarkers and breast cancer risk within the NHS II cohort [37]. Using incidence density sampling, women who did not have any type of cancer (other than non-melanoma skin cancer) at the time of the case’s cancer diagnosis (controls) were matched 1:2 with women diagnosed with in situ or invasive breast cancer (cases) on age at the time of blood collection, menopausal status and postmenopausal hormone use (current versus not current) at blood draw, day/time of blood draw, race/ethnicity and day in the luteal phase [38]. Our analysis included controls from this nested case-control study as well as additional eligible women within this cohort (without a history of any cancer other than non-melanoma skin) who were not included in the original nested breast cancer case-control study. We attempted to obtain mammograms closest to the time of blood collection (or approximately 1997 for those who did not provide blood samples). From all eligible women, 2590 provided consent and had a usable mammogram for density estimation. From the entire cohort only 41 % of women completed a food frequency questionnaire about diet during high school, and of 1874 premenopausal women with available mammograms, only 743 had data on adolescent fiber intake and essential covariates and were included in the analysis. This study was approved by the Institutional Review Board at the Brigham and Women’s Hospital. Consent was obtained or implied by return of questionnaires.

### Dietary assessment

Usual dietary intake and alcohol consumption during the past year were assessed with a semi-quantitative food frequency questionnaire (FFQ) with approximately 130 items which was included in the 1991, 1995, 1999, 2003, and 2007 questionnaires [39]. Responses were recorded in nine categories of intake frequency ranging from “never or less than once per month” to “six or more per day” for given portion sizes. Nutrient intakes were calculated by multiplying the frequency of consumption of each item by the nutrient content of the specified portions and then summing across all items, as previously described [39]. Every 4 years, the food composition database was updated to account for changes in the food supply. In 1997, participants were asked if they would be willing to complete a supplemental food frequency questionnaire about diet during high school (HS-FFQ). From the entire cohort, 56,928 women (49 %) indicated willingness to complete the HS-FFQ, and of those 47,355 (83 %) women returned the HS-FFQ in 1998. Food intake during adolescence was measured using a 124-item HS-FFQ, which was specifically designed to contain foods that were usually consumed during the periods from 1960 to 1980 when these women would have been in high school. Food items included in the food frequency questionnaire for adolescents and response categories were similar to those in the food frequency questionnaire for adults. Previous studies demonstrated high reproducibility of the nutrient intake estimates from these FFQs [40].

We used energy-adjusted intake estimates in all the analyses. Total fiber intake, fiber from fruit, vegetables, cereal, and legumes were defined as quartiles based on the distribution in the study sample (total fiber: ≤17.32, 17.33–20.40, 20.41–23.37, >23.37 g/day; fiber from fruit: ≤2.7, 2.8–4.2, 4.3–5.9, >5.9 g/day; fiber from vegetables: ≤4.4, 4.5–6.0, 6.1–7.9, >7.9 g/day; fiber from cereal: ≤4.4, 4.5–5.5, 5.6–6.8, >6.8 g/day; fiber from legumes: ≤1.6, 1.7–2.3, 2.4–3.3, >3.3 g/day). Total nut intake included intake of peanuts and other nuts and categories were <1 time/month, 1–3 times/month, 1 time/week, and ≥2 times/week.

### Assessment of mammographic breast density

Mammographic breast density was assessed in three batches approximately 2–3 years apart. To quantify breast density, the craniocaudal views of both breasts for the first two batches of mammograms in the NHS II were digitized at 261 μm per pixel with a Lumisys 85 laser film scanner (Lumisys, Sunnyvale, CA, USA). The third batch of NHS II mammograms was digitized using a VIDAR CAD PRO Advantage scanner (VIDAR Systems Corporation; Herndon, VA, USA) and comparable resolution of 150 dots per inch and 12-bit depth). The Cumulus software (University of Toronto, Toronto, ON, Canada) was used for computer-assisted determination of the absolute dense area, non-dense area, and percent mammographic density on all mammograms [3, 41]. All NHS II images were read by a single reader. Although within-batch reproducibility was high (intraclass correlation coefficient ≥0.90) [7], density measures varied across the NHS II batches. We included a small subset of identical mammograms in all batches to account for batch drift in density measurement readings. The density measures from the second and third batches of NHS II mammograms were adjusted to account for the batch effect (whether due to intra-reader variability or scanner), as previously described [42]. Additionally, to assess the potential variability in percent density by scanner, we conducted a pilot study of 50 mammograms. These mammograms were scanned using both the Lumysis 85 laser scanner and the VIDAR CAD PRO Advantage scanner; percent density was measured by the same observer using Cumulus. The correlation between percent density as measured by the two scanners was 0.88.

Percent breast density was measured as percentage of the total area occupied by epithelial/stromal tissue (absolute dense area) divided by the total breast area. Because breast densities of the right and left breast for an individual woman are strongly correlated [41], the average density of both breasts was used in this analysis.

### Covariate information

Information on breast cancer risk factors was obtained from the biennial questionnaires closest to the date of the mammogram. For exclusion from this analysis, women were considered to be postmenopausal if they reported: (1) no menstrual periods within the 12 months before blood collection with natural menopause, (2) bilateral oophorectomy, or (3) hysterectomy with one or both ovaries retained, and were 54 years or older for ever smokers or 56 years or older for never smokers [43, 44].

### Statistical analysis

We used generalized linear regression to examine the associations of total fiber intake; fiber from fruits, vegetables, legumes, and cereal; and food sources of fiber (fruits, vegetables, and nuts) with percent density, absolute dense and non-dense areas, while taking into account the correlation between matched controls [45]. Because absolute dense and non-dense area measures were non-normally distributed, we used square root transformation to improve normality in all the regression analyses. Percent breast density did not require transformation. The lowest fiber intake category was used as the reference. The regression estimates were adjusted for age (continuous), body mass index (continuous), body mass index at age 18 (continuous), age at menarche (<12, 12–13, >13 years), parity and age at first child’s birth (nulliparous, 1–4 children with age at first birth <25 years, 1–4 children with age at first birth of 25–29 years, 1–4 children with age at first birth of ≥30 years, ≥5 children with age at first birth of <25 years, or ≥5 children with age at first birth of ≥25 years), a confirmed history of benign breast disease (yes, no), a family history of breast cancer (yes, no), alcohol consumption (0, 0– < 5, ≥5 g/day, unknown), average consumption of the same nutrient in adulthood (quartiles), caloric intake in adolescence (continuous), and average caloric intake in adulthood (continuous). Average nutrient and caloric intake in adulthood were calculated as the average of the estimates from all available FFQs administered before the date of the mammogram. In a secondary analysis, the regression estimates for nuts were additionally adjusted for adolescent total fiber intake. Finally, we calculated the mean intake of total fiber and nuts from the adolescent and adult intake estimates and explored the association of these average intake estimates with breast density measures in a sensitivity analysis.

To assess the overall trend for each of the types of the fiber intake, we used respective medians within each category. For nut consumption, the intake frequencies were first converted into servings and then the median servings in each quartile were used in the trend analysis. Statistical significance in all the analyses was assessed at 0.05 level. The analyses were performed using SAS software (version 9.2, SAS Institute, Cary, NC, USA).

## Results

In this study of 743 cancer-free premenopausal women, the average age at the mammogram was 44.9 years (range 34.0–55.0). Women in the highest total fiber intake quartile had a mean percent density of 41.2 %, mean absolute dense area of 94.4 cm^2^, and mean non-dense area of 146.2 cm^2^ as compared to 40.8 %, 90.9 cm^2^, and 144.7 cm^2^ in women from the lowest quartile, respectively. Distributions of breast cancer risk factors by the quartiles of total fiber intake are presented in Table 1. Women in the highest quartile of adolescent total fiber intake as compared to the lowest quartile had higher adult caloric and total fiber intake (1894 vs. 1732 calories and 21.6 vs. 17.1 g/day, respectively). There was a greater proportion of nulliparous women and women with a positive family history of breast cancer in the highest quartile of adolescent fiber intake as compared to the lowest quartile. Distribution of other risk factors did not differ by the adolescent total fiber intake.

**Table 1:** Descriptive characteristics of the study population (n = 743), by quartile of adolescent total fiber intake

Characteristic	Adolescent total fiber intake quartile			
	1^st^	2^nd^	3^rd^	4^th^
	(≤17.32 g/day, median 15.7) n = 183	(17.33–20.40 g/day, median 18.9) n = 184	(20.41–23.37 g/day, median 21.6) n = 190	(>23.37 g/day, median 26.5) n = 186
Mean (STD)				
Age at mammogram, years	45.2 (4.2)	44.9 (4.0)	45.0 (4.1)	44.6 (4.0)
BMI at mammogram	25.9 (6.0)	25.2 (5.6)	25.2 (4.7)	25.6 (6.5)
BMI at age 18	21.1 (3.0)	20.9 (2.7)	20.9 (2.8)	21.3 (3.3)
Percent density	40.8 (18.3)	41.5 (17.5)	41.2 (18.6)	41.2 (18.0)
Absolute dense area, cm^2^	90.9 (48.0)	94.3 (50.0)	96.3 (52.0)	94.4 (50.3)
Non-dense area, cm^2^	144.7 (78.4)	141.9 (76.8)	147.5 (78.2)	146.2 (79.2)
Age at menarche, years	12.4 (1.4)	12.3 (1.4)	12.4 (1.5)	12.4 (1.6)
Caloric intake, adolescence	2730 (848)	2672(702)	2857 (773)	2800 (768)
Caloric intake, adult^a^	1732 (453)	1820 (445)	1858 (467)	1894 (531)
Alcohol consumption, g/day	4.1 (6.0)	4.6 (8.7)	4.8 (8.5)	4.6 (7.3)
Adult total fiber intake, median (range)^b^	17.1 (8.1–35.3)	18.0 (5.9–39.5)	19.2 (8.4–48.4)	21.6 (12.3–43.6)
Number (%)				
Parity/age at first child’s birth				
Nulliparous	31 (16.9)	34 (18.5)	34 (17.9)	47 (25.3)
1–4 children, <25 years	57 (31.2)	54 (29.4)	50 (26.3)	42 (22.6)
1–4 children, 25–29 years	60 (32.8)	66 (35.9)	64 (33.7)	63 (33.9)
1–4 children, ≥30 years	34 (18.6)	28 (15.2)	40 (21.1)	32 (17.2)
≥5 children, <25 years	1 (0.6)	1 (0.5)	2 (1.1)	2 (1.1)
≥5 children, ≥25 years	0	1 (0.5)	0	0
History of biopsy-confirmed benign breast disease	34 (18.6)	37 (20.1)	33 (17.4)	32 (17.2)
Family history of breast cancer	13 (7.1)	16 (8.7)	17 (9.0)	19 (10.2)

*Abbreviations*: *BMI* body mass index, *STD* standard deviation

^a^Difference across adolescent total fiber intake quartiles statistically significant at 0.01 level (analysis of variance for continuous variables or chi-square test for categorical variables)

^b^Difference across adolescent total fiber intake quartiles statistically significant at 0.001 level (analysis of variance for continuous variables or chi-square test for categorical variables)

In multivariable analyses, total fiber intake during adolescence was not associated with percent breast density (*p* trend = 0.64), absolute dense area (*p* trend = 0.80), or non-dense area (*p* trend = 0.75) (Table 2). We found a marginal association of adolescent nut intake with absolute non-dense breast area only (*p* for trend = 0.05); however, this association was lacking a clear pattern (β = 0.06, 0.62 and 0.35 for 1–3 times/month, 1 time/week, and ≥2 times/week, respectively). The results were similar with additional adjustment for adolescent total fiber intake. Neither specific types of fiber nor major sources of fiber intake (fruits, vegetables, legumes, or cereal) during adolescence were associated with any of the mammographic density phenotypes (Table 2). Finally, we explored the association of the average consumption of total fiber and nuts between adolescence and adulthood with breast density; we found no statistically significant associations (data not shown).

**Table 2:** Association of adolescent fiber intake with premenopausal breast density (coefficient and 95 % confidence interval) (n = 743)

Type of fiber/food group	Percent density (untransformed)			Absolute dense area (square root- transformed)		Non-dense area (square root-transformed)	
	N	Age/BMI adjusted	Fully adjusted^a^	Age/BMI adjusted	Fully adjusted^a^	Age/BMI adjusted	Fully adjusted^a^
Total fiber, quartile (quartile median)							
1^st^ (15.7)	183	Reference	Reference	Reference	Reference	Reference	Reference
2^nd^ (18.9)	184	-0.51 (-3.62, 2.60)	-0.66 (-3.62, 2.31)	0.13 (-0.38, 0.63)	0.00 (-0.50, 0.49)	0.16 (-0.34, 0.65)	0.09 (-0.40, 0.57)
3^rd^ (21.6)	190	-0.74 (-3.83, 2.35)	-1.04 (-4.30, 2.22)	0.22 (-0.28, 0.72)	0.10 (-0.42, 0.61)	0.36 (-0.13, 0.85)	0.34 (-0.17, 0.85)
4^th^ (26.5)	186	-0.19 (-3.29, 2.92)	-0.82 (-4.16, 2.53)	0.14 (-0.37, 0.64)	-0.09 (-0.61, 0.44)	0.16 (-0.33, 0.66)	0.06 (-0.49, 0.61)
*p* trend		0.92	0.64	0.59	0.80	0.48	0.75
Fiber from fruits, quartile (quartile median)							
1^st^ (1.9)	193	Reference	Reference	Reference	Reference	Reference	Reference
2^nd^ (3.5)	184	-0.10 (-3.18, 2.97)	-0.71 (-3.73, 2.31)	0.00 (-0.49, 0.50)	-0.01 (-0.54, 0.53)	0.07 (-0.42, 0.56)	0.23 (-0.27, 0.73)
3^rd^ (5.0)	184	-0.65 (-3.72, 2.42)	-2.11 (-5.28, 1.05)	-0.18 (-0.68, 0.31)	-0.30 (-0.80, 0.20)	0.01 (-0.48, 0.50)	0.21 (-0.30, 0.73)
4^th^ (7.2)	182	-0.01 (-3.09, 3.07)	-1.96 (-5.29, 1.38)	0.03 (-0.47, 0.53)	-0.26 (-0.80, 0.29)	0.11 (-0.38, 0.60)	0.29 (-0.28, 0.86)
*p* trend		0.94	0.21	0.95	0.24	0.72	0.36
Fiber from vegetables, quartile (quartile median)							
1^st^ (3.4)	187	Reference	Reference	Reference	Reference	Reference	Reference
2^nd^ (5.2)	190	-0.33 (-3.40, 2.74)	-0.15 (-3.17, 2.86)	0.20 (-0.29, 0.70)	0.22 (-0.27, 0.70)	0.18 (-0.31, 0.67)	0.11 (-0.37, 0.58)
3^rd^ (6.8)	180	-0.59 (-3.70, 2.52)	0.64 (-2.40, 3.69)	0.23 (-0.28, 0.73)	0.36 (-0.16, 0.87)	0.36 (-0.14, 0.85)	0.19 (-0.30, 0.68)
4^th^ (9.8)	186	0.36 (-2.72, 3.44)	1.05 (-2.21, 4.32)	0.25 (-0.25, 0.75)	0.29 (-0.24, 0.82)	0.15 (-0.34, 0.64)	-0.02 (-0.56, 0.53)
*p* trend		0.80	0.46	0.37	0.31	0.54	0.91
Fiber from legumes, quartile (quartile median)							
1^st^ (1.2)	201	Reference	Reference	Reference	Reference	Reference	Reference
2^nd^ (2.0)	186	-2.75 (-5.58, 0.09)	-2.92 (-5.72, -0.12)	-0.13 (-0.61, 0.36)	-0.14 (-0.61, 0.34)	0.29 (-0.18, 0.77)	0.34 (-0.13, 0.81)
3^rd^ (2.7)	177	0.40 (-2.73, 3.53)	-0.31 (-3.55, 2.93)	0.15 (-0.34, 0.65)	0.10 (-0.41, 0.60)	0.01 (-0.50, 0.53)	0.14 (-0.40, 0.68)
4^th^ (4.5)	179	-0.02 (-3.02, 2.97)	0.20 (-2.94, 3.34)	0.16 (-0.33, 0.65)	0.17 (-0.34, 0.68)	0.09 (-0.39, 0.57)	0.04 (-0.46, 0.54)
*p* trend		0.56	0.49	0.37	0.38	0.97	0.83
Fiber from cereal quartile (quartile median)							
1^st^ (3.8)	194	Reference	Reference	Reference	Reference	Reference	Reference
2^nd^ (5.0)	173	-0.14 (-3.18, 2.90)	-0.90 (-3.92, 2.12)	-0.10 (-0.60, 0.40)	-0.23 (-0.74, 0.27)	-0.11 (-0.59, 0.37)	-0.04 (-0.52, 0.44)
3^rd^ (6.1)	203	0.67 (-2.31, 3.65)	0.48 (-2.51, 3.47)	0.10 (-0.38, 0.59)	0.08 (-0.41, 0.57)	-0.12 (-0.61, 0.37)	-0.08 (-0.58, 0.42)
4^th^ (7.9)	173	-1.02 (-4.00, 1.97)	-1.84 (-4.86, 1.18)	-0.20 (-0.71, 0.30)	-0.29 (-0.80, 0.23)	-0.04 (-0.53, 0.45)	0.06 (-0.45, 0.57)
*p* trend		0.61	0.36	0.57	0.47	0.89	0.83
Total nuts, servings							
< 1/month (0 serv/day)	307	Reference	Reference	Reference	Reference	Reference	Reference
1–3/month (0.07 serv/day)	177	-1.00 (-3.81, 1.81)	-1.11 (-4.00, 1.77)	-0.18 (-0.64, 0.27)	-0.17 (-0.62, 0.29)	0.05 (-0.40, 0.50)	0.06 (-0.38, 0.50)
1/week (0.14 serv/day)	233	-1.07 (-3.66, 1.52)	-1.79 (-4.52, 0.94)	0.14 (-0.28, 0.56)	0.11 (-0.34, 0.57)	0.49 (0.08, 0.90)	0.62 (0.17, 1.07)
≥ 2/week (0.57 serv/day)	24	-1.54 (-7.89, 4.81)	-2.49 (-8.70, 3.71)	0.03 (-1.00, 1.06)	-0.06 (-1.20, 1.08)	0.23 (-0.78, 1.23)	0.35 (-0.37, 1.07)
*p* trend		0.48	0.27	0.72	0.90	0.18	0.05
Fruit intake, quartile (quartile median)							
1^st^ (0.6)	188	Reference	Reference	Reference	Reference	Reference	Reference
2^nd^ (1.2)	183	-0.39 (-3.29, 2.51)	-0.43 (-3.47, 2.61)	0.08 (-0.40, 0.56)	0.17 (-0.34, 0.68)	0.25 (-0.21, 0.71)	0.36 (-0.13, 0.84)
3^rd^ (1.8)	181	0.34 (-2.70, 3.38)	-0.30 (-3.66, 3.06)	0.13 (-0.34, 0.61)	0.19 (-0.35, 0.73)	0.15 (-0.34, 0.64)	0.38 (-0.15, 0.91)
4^th^ (2.7)	191	-0.18 (-3.18, 2.82)	-1.32 (-4.89, 2.26)	0.15 (-0.36, 0.66)	0.07 (-0.55, 0.68)	0.27 (-0.21, 0.75)	0.48 (-0.09, 1.06)
*p* trend		>0.99	0.75	0.55	0.88	0.36	0.14
Vegetable intake, quartile (quartile median)							
1^st^ (1.2)	186	Reference	Reference	Reference	Reference	Reference	Reference
2^nd^ (2.0)	185	-0.91 (-3.92, 2.10)	-0.16 (-3.31, 2.98)	-0.20 (-0.67, 0.27)	-0.12 (-0.62, 0.37)	0.14 (-0.33, 0.62)	0.05 (-0.46, 0.55)
3^rd^ (2.9)	185	-0.16 (-3.00, 2.67)	0.04 (-3.22, 3.31)	0.34 (-0.15, 0.84)	0.45 (-0.11, 1.01)	0.38 (-0.10, 0.86)	0.43 (-0.11, 0.97)
4^th^ (4.4)	187	-0.59 (-3.68, 2.49)	0.08 (-3.70, 3.86)	0.08 (-0.41, 0.57)	0.27 (-0.33, 0.87)	0.25 (-0.24, 0.75)	0.28 (-0.31, 0.86)
*p* trend		0.85	0.93	0.40	0.20	0.27	0.29

*Abbreviations*: *BMI* body mass index

^a^Adjusted for age (continuous), body mass index (continuous), body mass index at age 18 (continuous), age at menarche (<12, 12–13, >13 years), parity and age at first child’s birth (nulliparous, 1–4 children with age at first birth <25 years, 1–4 children with age at first birth of 25–29 years, 1–4 children with age at first birth of ≥30 years, ≥5 children with age at first birth of <25 years, or ≥5 children with age at first birth of ≥25 years), a confirmed history of benign breast disease (yes, no), a family history of breast cancer (yes, no), alcohol consumption (0, 0– < 5, ≥5 g/day, unknown), average consumption of the same nutrient in adulthood (quartiles), caloric intake in adolescence (continuous), average caloric intake in adulthood (continuous)

## Discussion

In this study of cancer-free premenopausal women, we investigated the associations of adolescent fiber intake with mammographic density, a strong and consistent predictor of breast cancer. Adolescent fiber intake was not associated with any of breast density phenotypes. While we noted a borderline significant positive association between total nut intake during adolescence and absolute non-dense breast area, the lack of clear pattern of association suggests that this could be a chance finding. Our findings contribute to the very limited evidence on the association of adolescent fiber intake and premenopausal breast density.

Some, but not all, previous studies have suggested that higher fiber intake may be associated with a reduced risk of breast cancer [46, 47]. A recent meta-analysis of prospective studies found an inverse association of total fiber with breast cancer risk (relative risk [RR] for high vs. low intake = 0.93, 95 % confidence interval [CI] 0.89–0.98 for total fiber), though the magnitude of this association was only modest [47]. Adolescent fiber intake has been suggested to reduce breast cancer risk in adulthood in some of the previous studies [16, 27, 48]. The results of an earlier study in NHS showed reduced risk of breast cancer in women with higher adolescent dietary fiber intake (RR for 5^th^ quantile vs. 1^st^ quantile = 0.78, *p* trend = 0.09) [27]. A recent analysis in the NHS II also showed a significant inverse association between adolescent fiber intake and breast cancer (RR = 0.81 for highest vs. lowest quintile; 95 % CI 0.72–0.91; *p* trend = 0.002) [17]. In the NHS II, high fiber and nut intake during adolescence was also associated with significantly lower risk of benign breast disease, another strong breast cancer risk factor (hazard ratio [HR] for highest vs. lowest quintile of adolescent fiber intake = 0.75, 95 % CI 0.59–0.96) [49].

Several biological mechanisms were suggested as a possible explanation for potential effects of dietary fiber on breast cancer risk, including its influence on circulating estrogen levels via inhibited intestinal reabsorption and increased fecal excretion as well as increased small intestine transit time leading to a slower glucose absorption and reduced insulin secretion [47]. On the other hand, a recent study reported a positive association between fiber intake and insulin-like growth factor 1 (IGF-I) concentrations (1.9 % increase per each standard deviation increase in intake) [50], which previously showed positive association with breast cancer risk [51] and mammographic breast density [52, 53].

Very limited data exists on associations of adolescent or childhood fiber intake and breast density. Among 1161 cancer-free women participating in a British cohort (Medical Research Council National Survey of Health and Development), there was no statistically significant association between low-fat/high-fiber dietary pattern at age 4 years and adult percent density evaluated with computer-assisted thresholding method [32]. A follow-up study among 230 participants in the Dietary Intervention Study in Children found no statistically significant associations of fiber intake at age 8–10 years with fibroglandular volume, measured with non-contrast MRI or the ratio of the fibroglandular volume over total volume of the breast at ages 25–29 [31]. Similarly, we found no statistically significant association of adolescent total fiber intake or fiber from specific sources and food groups with breast density.

Our study is the largest study to date that systematically investigated associations of adolescent fiber intake with mammographic breast density in premenopausal women. We examined, for the first time, the associations of adolescent fiber intake with absolute dense and non-dense area. In addition to the total fiber intake, we examined associations of specific sources of fiber and food groups with breast density. The analysis used data from the Nurses’ Health Study II, an established cohort with more than 25 years of follow-up, ascertainment of disease status, and comprehensive information on breast cancer risk factors and breast density. While we found no statistically significant associations, with n = 743, we had sufficient statistical power (>80 %) to detect even modest effects (e.g., 5–10 percentage points in average percent mammographic density, which translates into approximately 7 % change in breast cancer risk [6, 54]) thus making our null result informative.

Our study has a few limitations. The examined associations are based on the density measures from a single mammogram, which might not be reflective of the woman’s lifelong density pattern; however, studies have suggested that a single measure can predict breast cancer risk for up to 10 years in both pre- and postmenopausal women [6, 55]. Despite the prospective nature of the cohort, potential errors in recall of fiber intake, especially for adolescent diet, are possible, since women recalled their high school diet (on average 20–23 years from before the questionnaire date). However, previous studies suggest that recall of adolescent diet is reasonably reproducible and sufficiently precise to examine associations of adolescent diet with health outcomes in epidemiologic studies [56].

## Conclusions

We investigated the associations of adolescent fiber intake with percent density, absolute dense and non-dense areas in premenopausal women. Our findings do not support the hypothesis that adolescent fiber intake is associated with premenopausal mammographic density. If observed associations with breast cancer risk are causal, then the effect may not be mediated through mammographic density.

## Abbreviations

BMI: Body Mass Index
CI: Confidence Interval
FFQ: Food Frequency Questionnaire
HS-FFQ: High School Food Frequency Questionnaire
NHS II: Nurses’ Health Study II
RR: Relative Risk
